# Cell toxicity mechanism and biomarker

**DOI:** 10.1186/s40169-018-0212-7

**Published:** 2018-10-29

**Authors:** Yong Zhang

**Affiliations:** Zhongshan Hospital Institute of Clinical Science, Fudan University, Shanghai Institute of Clinical Bioinformatics, Biomedical Research Center, Shanghai, China

**Keywords:** Cell toxicity, NO, ROS, Oxidative stress, Mitochondrion dysfunction, Nanoparticle, Biomarker

## Abstract

Cell toxicity may result in organ dysfunction and cause severe health problem. Recent studies revealed many toxicants may induced the over production of Nitric oxide, reactive oxygen species and the subsequent oxidative stress, cause cell toxicity. Mitochondrion dysfunction maybe the subsequent consequence of oxidative stress and has been recognized as another contributing factor in cell toxicity. Besides, oxidative products induced by some toxicants may also produce the compounds that damage cell DNA, leading to toxicity. Especially, the significance of nanoparticle induced cell toxicity was disclosed recently and attract more concern. The mechanism mainly includes inflammation, oxidative stress and DNA damage. On the other side, some biomarkers of cell toxicity including autophagy, cytokines, miRNA has been identified. The understanding of these phenomenon may enable us to clarify the cell toxicity mechanism then contribute to cell toxicity protection, disease treatment and drug side effect prevention.

## Introduction

Cell toxicity is caused by exogenous toxicant which can damage cells, especially when the toxicant can cause cell death and serious organ dysfunction [1]. The effects of a toxicant are usually dose-dependent and species–specific. Cell toxicant include chemical agent, environment pollutant, natural plants extract and pharmaceutical drugs [2–4]. The mechanism of cell toxicity has been investigated in several decades and still attract interest of today.

The mechanisms of cell toxicity are widely involved. It has long been proved that toxicant may induce overproduction of Nitric oxide (NO), reactive oxygen species (ROS) and the subsequent oxidative stress [5]. The high level of NO, ROS and the subsequent oxidative burst have been identified as one main mechanism of severe cell toxicity or even organ dysfunction. Besides, mitochondrion dysfunction was also noticed as a consequence of oxidative stress, especially in neuro cells toxicity [6–8]. Toxicity agents may also induce and release compounds that directly damage DNA, causing cell apoptosis and toxicity [9–11].

Oxide nanoparticles (NP) induced cell toxicity has been gradually revealed and attract significant attention as their great application in medicine. Nanoparticles are particles between 1 and 100 nm (nm) in size and may be absorbed by human cells and may increase the occupational and public exposure and yield extraordinary hazards for human health. NPs in human cells may cause a serials of cell physiological change, ultimately resulted in cell toxicity [12, 13]. Some studies showed exposure to NPs induced cell toxicity were inflammation involved, such as IL-8 [14, 15].

These recent discovered cell toxicants and their toxicity mechanism may enable us to alleviate the cell dysfunction and promote cell protection. Here we review these articles about cell toxicity, its toxic mechanism and the associated biomarkers, try to summarize the recent progress and hotspot in this area and found some potential therapy target in cell toxicity prevention.

## NO overproduction

**Fig. 1:**
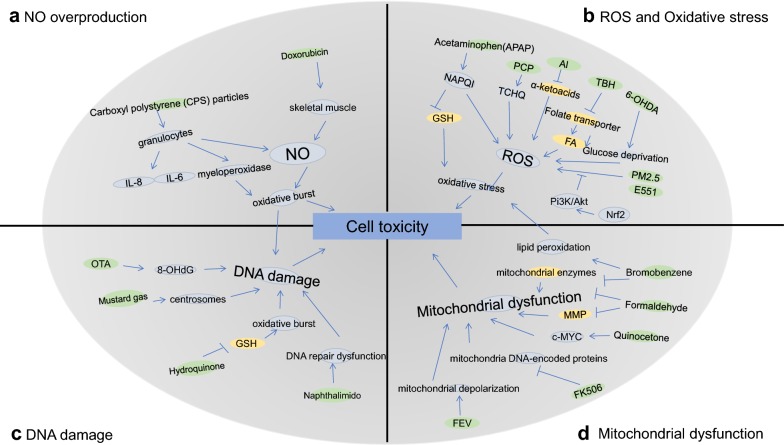
The mechanism of cell toxicity mainly include: **a** NO or cytokines overproduction induced oxidative burst, **b** ROS and oxidative stress, **c** DNA damage, **d** Mitochondrial dysfunction. Toxicant were shown as green

**Fig. 2:**
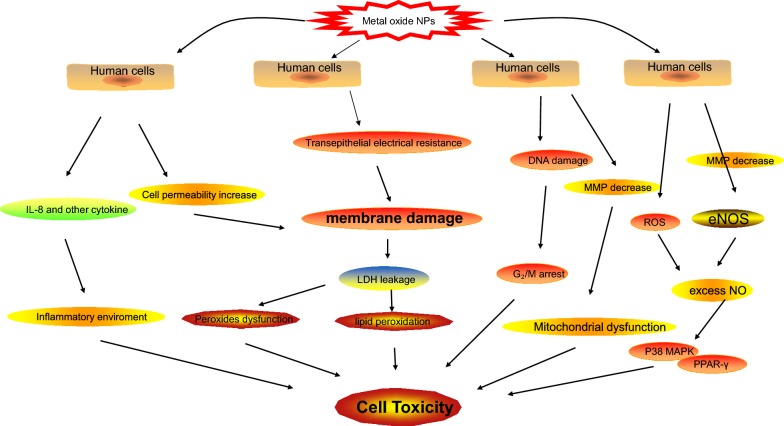
Mechanism of Metal oxide NPs induced cell toxicity. Include inflammatory environment, membrane damage, excess NO and Mitochondria dysfunction

NO is believed to play duel role in cell function, either detrimental or protective. NO regulation plays a critical role in cell function, especially in endothelial cells. NO may regulate the normal vascular tone and the repression of NO may cause proatherogenic situation [16, 17]. However, NO overproduction may also cause cell dysfunction or even cell toxicity. The high level of NO and related oxidative burst have been identified as one main mechanism of several cell toxicity. For instance, doxorubicin may induce skeletal muscle dysfunction and cardiotoxicity through NO. The administration of doxorubicin may cause the increased intracellular and interstitial NO concentrations in the SM, leading to SM dysfunction and myocardial cell toxicity (Fig. 1) [18]. NO may also be involved in NPs included oxidative burst of granulocytes and impair phagocyte function. The high concentrations of carboxyl polystyrene particles may stimulate myeloperoxidase release of granulocytes and NO over production in macrophages, cause cell toxicity [19]. MgNPs also has cell toxicity in a normal biological system. MgNPs may impair the proliferation of human umbilical vein vascular endothelial cells. MgNPs exposure may increase the activity of endothelial NO synthase, cause the over production of NO and leading to cell toxicity (Fig. 2) [20].

Several pathways may involve in NO overproduction induced cell toxicity. NO may suppress NF-κB pathway, potentiates TNF-α induced neurotoxicity [21]. NO may also activate p38 MAPK and p53 pathways, further increase DNA double strand breaks in microglia. However, the activation of both Akt and ERK cascades may alleviate the DNA damages induced by NO [22]. In diabetes and Alzheimer’s disease, the upregulates of NO synthase was also identified with the involved pathway of PPAR-γ, P38. These signaling changes are blocked by PPAR-γ small-interfering RNA transfection, and is also blocked by the NO inhibitor and p38 inhibitor [23].

## ROS and oxidative stress

ROS was identified as a pivotal modulator factor in immune system, neural system, infection and cancer development [24–27]. ROS homeostasis is essential in the sustain of cells function. Toxicity agents induced the over production of ROS and the subsequent oxidative stress may cause severe cell toxicity.

Researches have revealed ROS was involved in cell toxicity induced by variety of exogenous toxicant. For instance, acetaminophen is a well-known liver toxicant, acetaminophen-treated liver cells showed the significant increased ROS production and glutathione depletion, cause hepatocytes toxicity and death [28]. Pentachlorophenol, a pesticides, may cause high toxicological impact in hepatocytes with the involvement of ROS overproduction and oxidative stress. Antioxidants such as ascorbic acid and quercetin may modulate the toxicity effects of Pentachlorophenol [29]. Metal pollutants such as Aluminum may generate intracellular ROS and triggers a metabolic shift towards lipogenesis in astrocytes and hepatocytes. ROS activation is associated with impaired mitochondrial activity, anaerobiosis and the channeling of α-ketoacids towards anti-oxidant defense. These process leads to a reduction in ATP synthesis, were potential cause of brain and liver disorders [30]. The ROS induced cell toxicity have been revealed by temporal imaging. Cytoplasmic sensor in neuroblastoma cells demonstrated the 6-hydroxydopamine may induced oxidative stress and glucose deprivation enhanced ROS, cause neuroblastoma cells toxicity (Fig. 1) [31].

Cell toxicity of particulates intake in human cells has attract concern recently. Especially, airborne particulate with a diameter less than 2.5 μm (PM2.5) in considered to be a main cancerogen for lung cancer. A study revealed an increase of intracellular ROS with a time-dependent manner when human type II alveolar epithelial A549 cells exposed to PM2.5 particles. PM2.5 induced ROS may activate Nrf2-mediated defenses, such as HO-1 expression, against oxidative stress through PI3K/AKT signaling pathway [32]. Another nanoparticle, silica, is commonly used in food products, also revealed cell toxicity problem. High concentration of silica may cause significant cytotoxic effects on human lung fibroblast cell. Silica may induces a dose-dependent cell toxicity and elevated ROS levels, and meanwhile trigger the protective gene expression levels of stress-responsive genes (CAT, GSTA4, TNF, CYP1A, POR, SOD1, GSTM3, GPX1, and GSR1), which may be the potential therapy medicine for lung fibrosis [33]. AgNPs used in medical supplies may cause cytotoxic effect by ROS with decreased activities of superoxide dismutase and glutathione peroxides and the increased level of LPO [34]. Iron oxide nanoparticles also have potential toxicity by disrupt the barrier function of epithelium. Human placental cell line BeWo b30 was grown as epithelia and subsequently been assessed for epithelial integrity when exposure to α-Fe_2_O_3_ nanoparticles. Transepithelial electrical resistance indicated that exposure to the α-Fe_2_O_3_ nanomaterial resulted in leakiness of the epithelium and increases in cell death and ROS. Genotoxicity as assessed by DNA microarray and confirmed by QPCR indicated that the large diameter particles (78 nm) induce apoptosis in these cells (Fig. 2) [35].

## Mitochondrial dysfunction

Mitochondrion is critical in energy metabolism and playing key roles in biochemical synthesis, redox control and apoptosis. Alterations in mitochondrial function are increasingly being recognized as a contributing factor in many human diseases [36–39]. Recent understanding showed mitochondrion dysfunction and oxidative stress are usually co-existing in toxicant induced cell toxicity, and maybe a prevention method in cell toxicity [40].

Bromobenzene is a toxin may cause liver and kidney damage through oxidative stress. It may also induce mitochondrial dysfunction with the decreased activities of mitochondrial enzymes [41]. Formaldehyde induced toxicity is also with the involvement of oxidative stress and mitochondrial dysfunction. Formaldehyde may reduce mitochondrial membrane potential, inhibit mitochondrial respiratory enzymes such as NADH dehydrogenase (complex I), cytochrome c oxidase (complex IV), and promote cell apoptosis through initiator caspase-9 and apoptosis-effector caspase 3/7 [42]. Similarly, Quinocetone may triggers toxicity on HepG2 cells by oxidative stress and mitochondrial apoptotic. Quinocetone may reducing the activities of endogenous antioxidant enzymes and further promote apoptosis through c-MYC-dependent activation of the mitochondrial apoptotic pathway [43]. Toxicant cyclosporine A and cannabidiol may induce ROS in monocytic cell line. Impaired mitochondrial function was accompanied by elevated ROS and cell apoptosis level. Mitochondrial dysfunction may be also one mechanism of cytotoxicity in immune cells [44].

Some toxicant may directly induce mitochondrial dysfunction, result in cell apoptosis. For instance, FK506 is an important immunosuppressive medication and may provoke neurotoxicity and nephrotoxicity. FK506 may provokes an important decrease in oxygen consumption, reduction in the synthesis of mitochondria DNA-encoded proteins [45]. These mitochondrial dysfunction results are similar to those triggered by rapamycin immunosuppressive properties. Efavirenz induced mitochondrial depolarization may trigger mitochondrial morphology alteration and mitochondria mediated apoptosis [46]. It is also similar in the imatinib mesylate influences mitochondrial signaling leading to mitochondrial dysfunction and cardiotoxicity (Fig. 1) [47].

## DNA damage

DNA damage is another common mechanism of cell toxicity. Toxicant may induce compounds that damage DNA and un-repaired DNA damages may cause cell death. Besides, these types of alteration can be replicated and passed on to subsequent cell generations. These cell toxicities may regulate gene expression and change gene function, possibly contribute to progression to cancer.

Chratoxin A may induce DNA damage as a mechanism of cell toxicity. Chratoxin A treated BME-UV1 and MDCK epithelial cells showed a significant increase of cell apoptosis with the increased level of DNA integrity impairment [48]. Mustard gas is an alkylating agent that increases cell toxicity and the incidence of cancer with the mechanism of induce DNA damage. Mustard gas analog may induce centrosome amplification and chromosome instability in cells, which may hasten the mutation rate necessary for tumorigenesis [49]. Naphthalimido compounds may induced cell toxicity by intercalating to DNA via the major groove and induce DNA damage. A lower concentration of Naphthalimido may also induced a significant decrease in repair of DNA damage [50].

Besides, DNA damage may also be synergetic with oxidative stress to induce cell toxicity. Hydroquinone exposure in air pollution may cause genotoxicity on human lung cells. Chromosomal aberration and associated DNA damage was observed in hydroquinone treated lung epithelium cell A549, meanwhile an increase oxidized glutathione and the reduced anti oxidization glutathione was also observed (Fig. 1) [51]. In cell toxicity of silver nanoparticles, DNA damage and ROS may also have synergetic effect. It is proved by the co-existence of decreased mitochondrial membrane potential, cell G_2_/M phase arrest and the increased of anti-peroxide effect in AgNP exposure cells (Fig. 2) [34].

## Cell toxicity biomarker and potential therapy target

Autophagy is a phenomenon of cell toxicity, and may also be an effective marker in anti-cancer efficiency of cancer treatment. A study suggests that erlotinib combined with radiotherapy may remarkably induce apoptosis in lung cancer cells with provoked autophagy. The cell toxicity of lung cancer cells may be reversed by autophagy inhibitor chloroquine [52]. Besides, pinus radiata bark extract may induces cytotoxic effects in human breast cancer cells with increased accumulation of autophagic markers [53].

ROS is the marker and may be also the potential therapy target of cell toxicity. LPO, increase catalase activity and mitochondria metoclopramide groups are the markers of ROS and mitochondria associated cell toxicity. The extract of *Sambucus ebulus* L. fruit may protect neuro cells through relieve ROS in brain mitochondria. Increased plasma antioxidants or scavenging of free radicals were observed in the extract treated patients. The improvement of the two biomarkers may be therapy target of the restore of mitochondria function [54].

The increased cytokines may be the marker of cell toxicity in some inflammation associated diseases. For example, severe systemic inflammation may trigger lung cell toxicity Some cytokines group may be the marker for inflammation induced ARDS. These biomarkers including bone morphogenetic protein-15, CXCL16, CXCR3, IL-6, protein NOV homolog, glypican 3, IGFBP-4, IL-5, IL-5R alpha, IL-22 BP, leptin, MIP-1d, and orexin B. The overexpressed IL-6, CXCL16, or IGFBP-4 may also represent the severity of the disease [55]. Another example is renal allograft rejection. Some inflammation factors may provide diagnostic biomarkers for predicting cell toxicity induced rejection. Proteomic study found 33 inflammatory proteins and the protein–protein network analyzes suggest ICAM-1 is the main biomarker in chronic rejection [56].

MicroRNA profiling could be a useful tool for the discovery of cell toxicity biomarkers and target therapy genes. The neuronal toxicity of MeHgCl may characterized by the overexpression of a signature composed of five miRNAs (miR-302b, miR-367, miR-372, miR-196b and miR-141) that are known to be involved in the regulation of developmental processes and cellular stress response mechanisms [57]. Besides, miR-146a, the target gene of MCL1 which encoding an anti-apoptotic protein, is a marker of H2O2-induced PC12 cells toxicity. MCL1 may activate JAK/STAT signaling pathway and attenuated H_2_O_2_-induced cell toxicity [58].

## Conclusion

Cell toxicity induced by environment toxicant and medicines may be resulted in severe organ dysfunction and disease. Recent studies has revealed the main cell toxicity mechanism includes NO overproduction, ROS induced oxidative stress, mitochondrial dysfunction, DNA damage. Especially, the toxicity of metal oxide NPs has been recognized in the recent years. All of which may enable us to clarify the cell toxicity mechanism. However, the toxicity prevention methods require further explicated to prevent toxicity and contribute to disease treatment.
